# Corpus luteum hemorrhage with acquired hemophilia A: a case report and literature review

**DOI:** 10.1186/s12905-022-02000-9

**Published:** 2022-10-11

**Authors:** Xiaofei Xie, Shaoru Jiang

**Affiliations:** Department of Gynecology, Jieyang People’s Hospital, Tianfu Road No.107, Rongcheng District, Jieyang, 522000 Guangdong People’s Republic of China

**Keywords:** Corpus luteum, Bleeding disorder, Acquired hemophilia A, Hemoperitoneum, Conservative management

## Abstract

**Background:**

The rupture of the corpus luteum (CL) may occur at all stages of a woman’s reproductive life. Bleeding of the ruptured CL varies from self-limiting hemorrhage to massive hemoperitoneum, causing the shock and subsequent emergency surgery. But hemoperitoneum secondary to ruptured CL is a rare complication and situation for women with bleeding disorders.

**Case presentation:**

We here describe a case of severe CL hemorrhage with factor VIII deficiency. We chose conservative management instead of surgery for the abnormal hemostatic condition. With blood product and factor concentrate support, conservative management was successful in avoiding surgery in the episode of bleeding.

**Conclusion:**

Gynecologist should be alert for the patients with abnormal hemostatic condition. Selective patients presenting with CL hemoperitoneum association with bleeding disorders may undergo conservative management and avoid the risk of surgery.

## Background

Ovulation is a physiologic event monthly and may rarely be complicated by rupture of the CL. Spontaneous but self-limiting bleeding would fill the central cavity when the CL is formed during the luteal phase of the ovarian cycle. The CL cyst may form when the bleeding is rather excessive, and rupture when triggered by exercise, coitus or trauma. Clinical symptoms include the sudden onset of lower abdominal pain, peritoneal irritation by the blood effusion. The differential diagnosis includes ectopic pregnancy, adnexal torsion and pelvic inflammatory disease (PID). When the hemoperitoneum worsen, symptoms such as hypotension, syncope and cardiovascular collapse may appear. The ultrasound (US) technology has a key-role in the CL hemorrhage diagnosis. The appearance of a hemorrhagic ovarian cyst can be different in size, thickness of the cyst wall, and internal echo pattern depending on the formation and lysis of the clot. Usually, it appears as an unilocular round mass with well-defined, regular and thin walls. Conservative management like antifibrinolytic, liquid infusion, blood transfusions and antibiotic would be selected since the hemorrhagic CL is often self-limiting. Laparoscopy is preferred rather than laparotomy in most operative managements, with surgical options such as luteumectomy, ovarian wedge-shaped excision or oophorectomy [1].

The hemorrhagic CL may lead to little clinical consequence in women with normal hemostatic function. However, serious and even life-threatening episodes have been described in women with abnormal hemostatic condition, such as anticoagulants, hemophilia A and congenital afibrinogenemia [2]. Such hemorrhagic CL patients with abnormal hemostatic condition should be managed by gynecologist and hematologist. We herein present a rare but severe case of CL hemorrhage with FVIII deficiency, while conservative management brings a better outcome for this patient.

## Case presentation

A 27-year-old woman with acute onset lower abdominal pain for 2 days was transferred to our hospital by ambulance. She was diagnosed as idiopathic thrombocytopenic purpura (ITP) 2 years ago in another hospital based on abnormal vaginal bleeding in her first trimester. After treating with prednisone and intravenous immunoglobulin (IVIG) she got remission and delivered a healthy daughter later. After 3 months of delivery, dermal ecchymosis appeared on her arms and legs gradually and these symptoms were not completely relieved by the treatment of glucocorticoids as well as Chinese traditional medicine. No bleeding disorder was reported in her family history information. Physical examination showed mucocutaneous bleeding on arms and legs, a regular pulse of 115/min, a blood pressure of 116/69 mmHg and a temperature of 36.5 °C. There was abnormal tenderness in the lower left abdomen. Dark red blood was observed by culdocentesis test. Ultrasonography showed a left adnexal cystic mass of 61 mm × 25 mm with low level internal echoes and free liquid areas in the pelvis, bilateral iliac fossa, Morrison pouch and perisplenic space. The pregnancy test was negative. Her hemoglobin (Hgb) was 8.3 g L^−1^ and platelet count 53 × 10^9^ L^−1^. Her activated partial thromboplastin tine (aPTT) was 91.9 s while both prothrombin time (PT) and fibrinogen were normal. A diagnosis of hemorrhagic corpus luteum combined with ITP was made. The aPTT was 92.7 s and the Hgb fell to 6.8 g L^−1^ after transfused with 400 ml of fresh frozen plasma. Since the risk of surgical approach was extremely high as the aPTT was enormously prolonged, a conservative management was undertaken with strict vital monitoring and blood support. Drug therapy such as tranexamic acid (TA) and aminocaproic acid (EACA) were also used. Besides, we performed the coagulation factors tests under the consultation by hematologist and found that the activity of FVIII was significantly decreased to 0.10% (50–150) simultaneously the FVIII inhibitor was 5.16 BU (0–0.6). Her lupus anticoagulant testing was negative. She was then confirmed the diagnosis of acquired hemophilia A (AHA) and treated with the recombinant factor VIIIa (rFVIIIa). The Hgb fell to 5.3 g L^−1^ while the Platelet count increased to 97 × 10^9^ L^−1^ after 48 h of conservative management. After 3.5 U of blood and 800 ml of fresh frozen plasma transfusion, her condition began to improve in the following days. Ceftazidime injection was performed for 6 days after 72 h hospital admission since her temperature raised to 38.9 °C. The aPTT was 84.2 s after 5 days of rFVIIIa therapy. Laparoscopy surgery was not performed since the aPTT was still prolonged as well as her condition is improving. Ultrasonography after 2 weeks of conservative treatment showed that a left adnexal cystic mass of 99 mm × 65 mm with equal level internal echoes and ring blood flow. The hemoperitoneum showed a gradual resolution since there only left the pelvic liquid area which was 81 mm × 47 mm. In the meantime, her dermal ecchymosis on arms and legs faded away. In the end, she discharged in a good condition with her Hgb rose to 133 g L^−1^. Considering her unimproved prolonged aPTT under the treatment of rFVIIIa, we suggested her further consultation in hematology department.

## Discussion and conclusions

After ovulation, the follicle develops into CL and spontaneous bleeding may occur to form the corpus haemorrhagicum, which may later rupture. The complications of a ruptured CL range from a single CL hematoma to extensive hemoperitoneum, which may be life-threatening. Negative pregnancy test is important to exclude ruptured ectopic pregnancy. US is currently considered the gold standard technique for the diagnosis of CL hemorrhage. Most cases of ruptured CL with moderate hemoperitoneum can be treated conservatively. However, in the event of massive hemoperitoneum, even cardiovascular collapse, surgical treatment like laparoscopy is usually considered.

The bleeding of ruptured CL is often self-limited but may be more serious in women on anticoagulants or with a bleeding disorder. Coagulative disorders, which may also lead to abnormal uterine bleeding (AUB), require differential diagnosis which may cause impaired coagulative status[3]. Many cases of CL hemorrhage have been reported in patients with von Willebrand disease type 1, 2A, 3 [4–10], afibrinogenemia [11–14], Glanzmann’s thrombasthenia, hemophilia A [15], hemophilia B, deficiency of factor X, factor V and factor XIII, and in patients receiving anticoagulant therapy for antiphospholipid antibody syndrome (APS) [16–21]. However, we haven’t found any report about CL hemorrhage in patient with AHA (Table 1). Women with abnormal hemostatic condition also have a higher risk of ruptured CL, which recur in nearly 25–31% patients with long-term anticoagulation [22]. Silwer reported that 9 of 136 women with von Willebrand’s disease (VWD) had experienced CL hemorrhage [23]. In another report, consisted of 93 patients with severe FXIII deficiency, 4 of 20 (20%) women of reproductive age had experienced intraperitoneal bleeding at the time of ovulation [24]. If a pre-menopausal woman is on anticoagulants or has a personal or family history of a bleeding disorder, it is important to be aware of the possibility of CL hemorrhage when she suffers from an acute abdomen. Complete coagulation screening is essential for the identification of patients with bleeding disorders, anamnesis, anticoagulant therapy. In addition, family history can also provide important information [1]. Phenotype evaluation and molecular diagnosis will help a lot to distinguish different kinds of coagulative disorders [25].

**Table 1:** Reported cases of hemoperitoneum in patients with bleeding disorders

References	Diagnosis	Details	Management
Bottini et al. [10]	Type 3 VWD	Two patients had hemorrhagic CL	One had surgery (wedge resection of ovary), another treated with conservative management. Recurrence prevented by OC
Bottini et al. [10]	Afibrinogenemia	22 Years old. Three episodes of hemorrhagic CL	Each episode required surgery. Recurrence prevented by OC
Meschengieser et al. [9]	Combined mild VWD and mild storage pool defect	19 Years old. Three episodes of bleeding over 13 years	Surgery (wedge resection of ovary)
Ghosh et al. [8]	Type 3 VWD	29 Years old. Three episodes. Two of three needing resuscitation	Conservative management. Recurrence prevented by OC and TA
Gomez et al. [7]	Type 3 VWD	22 Years old. Hemoperitoneum from rupture of ovarian cyst	Exploratory laparotomy (oophorectomy)
Greer et al. [6]	Type 2 VWD mild	Hemorrhagic corpus luteum and broad ligament hematoma	Salpingo-oophorectomy while conservative management with cryoprecipitate had no effect
Jarvis et al. [5]	Type 1 VWD	Recurrent corpus hemorrhagicum two episodes	Blood support and exploratory laparotomy Conservative management No recurrence for 4 years by OC
Terzic et al. [4]	VWD severe	Massive hematoperitoneum caused by ovulation. Had right adnexectomy due to hemorrhagic CL four months prior	Conservative management by blood product and factor concentrate support. Recurrence prevented by OC
Cetinkaya et al. [13]	Congenital afibrinogenemia	24 Years old. Two episodes of massive intraabdominal bleeding due to ovulation	Exploratory laparotomy and excision of the ruptured follicle at first episode. Conservative management with blood support at second episode
Castaman et al. [12]	Congenital afibrinogenemia	24 Years old. Two episodes of hemoperitoneum during ovulation	Both episodes required operative intervention and replacement therapy. No recurrence over 5 years by OC
Koussi et al. [11]	Congenital afibrinogenemia	14 Years old. Developed intra-abdominal bleeding due to the rupture of an ovarian cyst	Replacement therapy (red cells and cryoprecipitate)
O’Brien et al. [15]	Hemophilia A	18 Years old. Hemorrhagic ovarian cyst	Conservative therapy (factor VIII therapy)
Dafapoulos et al. [19]	Factor X deficiency	24 Years old. Two episodes of hemoperitoneum from luteal cyst rupture	Both episodes removed the ruptured cyst by surgery
Khamashta et al. [18]	Antiphospholipid antibody syndrome	Severe ovarian hemorrhage during warfarin treatment	
Yamakami et al. [17]	Antiphospholipid syndrome	Three patients had severe hemorrhagic CL while receiving warfarin treatment	All of them required prompt blood transfusion and emergency surgery
Castellino et al. [16]	Antiphospholipid syndrome	Two episodes of ovarian hemorrhage while receiving oral anticoagulation	One required oophorectomy
Singh et al. [20]	Factor XIII deficiency	13 Years old. CL hemorrhage	Laparoscopic surgery with blood support
Badyal et al. [21]	Factor V deficiency	19 Years old. Recurrent CL hemorrhage three episodes	Each episode required surgery and blood support
Schneider et al. [14]	Congenital afibrinogenemia	22 Years old. Bleeding from a ruptured CL	Oophorectomy was performed with blood support

Surgical treatment is often the first choice when the CL rupture causes the massive hemorrhage or even cardiovascular collapse. Laparoscopy as a minimally invasive approach is usually preferred over laparotomy. The hospitalization of the patient is reduced with laparoscopy compared with laparotomy (55 ± 8 vs. 98 ± 14 h) and post-operative pain is significantly reduced [26]. Autologous blood transfusions using blood recovered from the peritoneal cavity should also be considered especially in the event of massive hemoperitoneum. However, surgical options such as ovarian wedge-shaped excision or oophorectomy would result in great impact on the ovarian function. Reversal of anticoagulant therapy may result in thromboembolism in the post-operative period and lead to increased morbidity. Besides, it would also increase the hemorrhage risk during surgery if the INR is higher than 3.0 or the abnormal hemostatic condition is not under control [27].

Conservative management includes cardiopulmonary support, close observation of vitals, antibiotics, correction of hemostatic condition with fresh frozen plasma and replacement of blood products. As a wait-and-see attitude, it applies for most cases of ruptured CL with moderate hemoperitoneum [26]. Gupta reported 3 cases on anticoagulation, 2 of which were treated with a successful conservative management by supportive measures and normalization of INR [28]. Payne described 3 patients presenting with hemoperitoneum were associated with factor VII deficiency, factor X deficiency and sitosterolemia. Recurrent episodes occurred in 2 of the patients with certain congenital bleeding disorders and conservative management with blood product and factor concentrate support was successful in avoiding surgery in 3 of the 5 episodes of bleeding [2]. The observational approach in hemodynamically stable patients could be the first-choice option in most cases [1].

In this case, we intended to choose laparoscopy first as the US and culdocentesis reminded us of the massive hemoperitoneum, but finally chose the conservative management when we found her with AHA and her aPTT was extremely abnormal. AHA, in contrast to congenital hemophilia, is a rare disease resulting from autoantibodies against autologous FVIII [29]. AHA incidence is estimated about 1–1.5 cases per million per year, which is most seen in adults who are older than 65 years of age. There is also a smaller peak in the incidence of AHA in young women (20–40 years) related to pregnancy. Almost half of the cases are idiopathic. The others are related to autoimmune diseases, malignancies, pregnancy, infections or medications [10–12]. This patient may have already developed AHA before she had dermal ecchymosis on arms and legs gradually 3 months later after delivery. It is called the pregnancy-related AHA [30–32]. But it didn’t cause the post-partum hemorrhage like other case which caused by thrombotic thrombocytopenic purpura (TTP) [33]. Options for first-line hemostatic agents include replacement therapy like rFVIIIa, bypassing agents like recombinant factor VIIa (rFVIIa) and other hemostatic approaches such as desmopressin (DDVAP) and TA. Human FVIII replacement, although effective in patients with low titers (< 5 BU), is not effective in patients with high titer inhibitor (> 5 BU) [32]. That may explain the unimproved prolonged aPTT in our case as her titer inhibitor was 5.16 BU before treatment. In addition, we should try rFVIIa and immunosuppression therapy (IST) like corticosteroid and cyclophosphamide as first-line therapies. Rituximab can also be considered as the second-line treatment and used in the case contraindicated to first-line therapies. IST achieves remission in about 60–80% of patients after a median of 5–6 weeks but requires close monitoring. By the way, IVIG has a limited role in the treatment of AHA. As a matter of fact, patients with AHA are best managed by, or in close consultation with, physicians experienced in AHA [30–32]. For the gynecologist, they should watch out for the patients with abnormal hemostatic condition. If there is close collaboration between hematologists and gynecologists, optimum management is more likely to be achieved and improved.

Patients with bleeding disorders or undergoing anticoagulant therapy have a higher risk of recurrence. The prevention of recurrence is desirable to avoid life-threatening bleeds and to preserve fertility. Numerous studies have investigated the effects of oral contraceptive (OC) pill on follicular cyst development and ovulation. In summary, current reports showed that OC resulted in the development of fewer follicular and correspondingly lutein cysts [2]. But we didn’t try such kind of precaution since our patient wish to conceive later. For the pregnancy-related AHA, the risk of recurrence in future pregnancy appears low, but patients should be aware of this possibility [31, 32].

In summary, rupture of CL is a common occurrence in women of reproductive age. Bleeding of the ruptured CL can vary from self-limiting hemorrhage to massive hemoperitoneum and may be more serious in women on anticoagulants or with a bleeding disorder. Management is based on patient characteristics, including the severity of symptoms, whether hemodynamic instability or not. Conservative management could be the preferred option, for selective patients presenting with CL hemoperitoneum associated with bleeding disorders. Gynecologist should be alert for the patients with bleeding disorders as the hemoperitoneum is severe, which consequently increases the risk of surgery. Close collaboration between hematologists and gynecologists is required. For women with bleeding disorders and without fertility desire, OC may be an appropriate precaution measure.

## Data Availability

The datasets used or analyzed during the current study are available from the corresponding author on reasonable request.

## Abbreviations

AHA: Acquired hemophilia A
APS: Antiphospholipid antibody syndrome
aPTT: Activated partial thromboplastin tine
AUB: Abnormal uterine bleeding
CL: Corpus luteum
DDVAP: Desmopressin
EACA: Aminocaproic acid
FVIII: Factor VIII
Hgb: Hemoglobin
IST: Immunosuppression therapy
ITP: Idiopathic thrombocytopenic purpura
IVIG: Intravenous immunoglobulin
OC: Oral contraceptive
PID: Pelvic inflammatory disease
PT: Prothrombin time
rFVIIIa: Recombinant factor VIIIa
rFVIIa: Recombinant factor VIIa
TA: Tranexamic acid
TTP: Thrombotic thrombocytopenic purpura
US: Ultrasound
VWD: Von Willebrand’s disease

## References

[1] Medvediev MV, Malvasi A, Gustapane S, Tinelli A (2020). Hemorrhagic corpus luteum: clinical management update. J Turk Soc Obstet Gynecol.

[2] Payne JH, Maclean RM, Hampton KK, Baxter AJ, Makris M (2007). Haemoperitoneum associated with ovulation in women with bleeding disorders: the case for conservative management and the role of the contraceptive pill. Haemophilia.

[3] Motta T, Laganà AS, Valenti G, la Rosa VL, Noventa M, Vitagliano A (2017). Differential diagnosis and management of abnormal uterine bleeding in adolescence. Minerva Ginecol.

[4] Terzic M, Likic I, Pilic I, Bila J, Knezevic N (2012). Conservative management of massive hematoperitoneum caused by ovulation in a patient with severe form of von Willebrand disease—a case report. Clin Exp Obstet Gynecol.

[5] Jarvis RR, Olsen ME (2002). Type I von Willebrand’s disease presenting as recurrent corpus hemorrhagicum. Obstet Gynecol.

[6] Greer IA, Lowe GD, Walker JJ, Forbes CD (1991). Haemorrhagic problems in obstetrics and gynaecology in patients with congenital coagulopathies. Br J Obstet Gynaecol.

[7] Gomez A, Lucia JF, Perella M, Aguilar C (1998). Haemoperitoneum caused by haemorrhagic corpus luteum in a patient with type 3 von Willebrand’s disease. Haemophilia.

[8] Ghosh K, Mohanty D, Pathare AV, Jijina F (1998). Recurrent haemoperitoneum in a female patient with type III von Willebrand’s disease responded to administration of oral contraceptive. Haemophilia.

[9] Meschengieser SS, Alberto MF, Salviú J, Bermejo E, Lazzari MA (2001). Recurrent haemoperitoneum in a mild von Willebrand’s disease combined with a storage pool deficit. Blood Coagul Fibrinolysis.

[10] Bottini E, Pareti FI, Mari D, Mannucci PM, Muggiasca ML, Conti M (1991). Prevention of hemoperitoneum during ovulation by oral contraceptives in women with type III von Willebrand disease and afibrinogenemia. Case reports. Haematologica.

[11] Koussi A, Economou M, Athanasiou-Metaxa M (2001). Intra-abdominal hemorrhage due to a ruptured corpus luteum cyst in a girl with congenital afibrinogenemia. Eur J Pediatr.

[12] Castaman G, Ruggeri M, Rodeghiero F (1995). Congenital afibrinogenemia: successful prevention of recurrent hemoperitoneum during ovulation by oral contraceptive. Am J Hematol.

[13] Cetinkaya SE, Pabuccu EG, Ozmen B, Dokmeci F (2011). Recurrent massive hemoperitoneum due to ovulation as a clinical sign in congenital afibrinogenemia. Acta Obstet Gynecol Scand.

[14] Schneider D, Bukovsky I, Kaufman S, Sadovsky G, Caspi E (1981). Severe ovarian hemorrhage in congenital afibrinogenemia. Acta Obstet Gynecol Scand.

[15] O’Brien PM, DiMichele DM, Walterhouse DO (1996). Management of an acute hemorrhagic ovarian cyst in a female patient with hemophilia A. J Pediatr Hematol Oncol.

[16] Castellino G, Cuadrado MJ, Godfrey T, Khamashta MA, Hughes GR (2001). Characteristics of patients with antiphospholipid syndrome with major bleeding after oral anticoagulant treatment. Ann Rheum Dis.

[17] Yamakami LYS, de Araujo DB, Silva CA, Baracat EC, de Carvalho JF (2011). Severe hemorrhagic corpus luteum complicating anticoagulation in antiphospholipid syndrome. Lupus.

[18] Khamashta MA, Cuadrado MJ, Mujic F, Taub NA, Hunt BJ, Hughes GR (1995). The management of thrombosis in the antiphospholipid-antibody syndrome. N Engl J Med.

[19] Dafopoulos K, Galazios G, Georgadakis G, Boulbou M, Koutsoyiannis D, Plakopoulos A (2003). Two episodes of hemoperitoneum from luteal cysts rupture in a patient with congenital factor X deficiency. Gynecol Obstet Invest.

[20] Singh N, Neeta S, Gupta N, Nupur G, Sarangi S, Shikha S (2008). Corpus luteal hemorrhage: an unusual manifestation of congenital factor XIII deficiency. Haemophilia.

[21] Badyal RK, Jain K, Mandrelle K, John MJ, Kakkar N, Bose SK (2015). Recurrent hemoperitoneum secondary to haemorrhage from the corpus luteum unmasks factor V deficiency. Blood Coagul Fibrinolysis.

[22] Faraj R, Martindale E, Hill S (2008). Massive ovarian cyst haemorrhage with haemoperitoneum as a complication of long-term anticoagulation. J Obstet Gynaecol.

[23] von Silwer J (1973). Willebrand’s disease in Sweden. Acta Paediatr Scand Suppl.

[24] Lak M, Peyvandi F, Ali Sharifian A, Karimi K, Mannucci PM (2003). Pattern of symptoms in 93 Iranian patients with severe factor XIII deficiency. J Thromb Haemost.

[25] Menegatti M, Palla R (2020). Clinical and laboratory diagnosis of rare coagulation disorders (RCDs). Thromb Res.

[26] Chen L, Ding J, Hua K (2014). Comparative analysis of laparoscopy versus laparotomy in the management of ovarian cyst during pregnancy. J Obstet Gynaecol Res.

[27] Itskovitz J, Brandes JM, Urbach J, Fisher M (1982). Ovarian hematoma and hemoperitoneum complicating anticoagulant therapy. Int Surg.

[28] Gupta A, Gupta S, Manaktala U, Gupta MM, Solanki V (2015). Conservative management of corpus luteum haemorrhage in patients on anticoagulation: a report of three cases and review of literature. Arch Gynecol Obstet.

[29] Elezovic I (2010). Acquired haemophilia syndrome: pathophysiology and therapy. Srp Arh Celok Lek.

[30] Franchini M, Vaglio S, Marano G, Mengoli C, Gentili S, Pupella S (2017). Acquired hemophilia A: a review of recent data and new therapeutic options. Hematology.

[31] Charlebois J, Rivard GÉ, St-Louis J (2018). Management of acquired hemophilia A: review of current evidence. Transfus Apheres Sci.

[32] Kruse-Jarres R, Kempton CL, Baudo F, Collins PW, Knoebl P, Leissinger CA (2017). Acquired hemophilia A: updated review of evidence and treatment guidance. Am J Hematol.

[33] Laganà AS, Sofo V, Salmeri FM, Chiofalo B, Ciancimino L, Triolo O (2015). Post-partum management in a patient affected by thrombotic thrombocytopenic purpura: case report and review of literature. Clin Exp Obstet Gynecol.

